# Real‐World Use of Polynucleotide Injections in Scar Prevention and Management: A Nationwide Survey of Korean Society for Anti‐Aging Dermatology (KAAD) Dermatologists

**DOI:** 10.1111/jocd.70887

**Published:** 2026-05-05

**Authors:** Nark Kyoung Rho, Kwang Ho Han, Boncheol Leo Goo, Hei Sung Kim

**Affiliations:** ^1^ Leaders Aesthetic Laser and Cosmetic Surgery Center Seoul Korea; ^2^ Nature Dermatology Clinic Seoul Korea; ^3^ Naeum Dermatology Clinic Seoul Korea; ^4^ Incheon St. Mary's Hospital The Catholic University of Korea Seoul Korea

**Keywords:** Korea, polynucleotide, real‐world practice patterns, scar management

## Abstract

**Background:**

Scarring is a common dermatologic concern with both aesthetic and functional implications. Polynucleotide (PN)‐based injectables have emerged as medical devices in dermatologic practice; however, real‐world evidence on their use in scar management remains limited.

**Objective:**

To describe current clinical practices, application patterns, and perceived roles of PN injections in scar prevention and management among dermatologists belonging to the Korean Society for Anti‐Aging Dermatology (KAAD).

**Methods:**

A nationwide, cross‐sectional, web‐based survey was conducted among KAAD dermatologists. The questionnaire assessed PN use by scar type and etiology, injection techniques, combination strategies, and barriers to adoption. Data were analyzed descriptively.

**Results:**

Among 501 respondents, 73.0% reported using PN for scar management. PN was most commonly used for scar prevention (81.4%) and atrophic scars (74.9%), with limited use in hypertrophic scars (10.1%). Intradermal injection over three sessions was the most common approach. PN was frequently incorporated into multimodal protocols, including botulinum toxin, fillers, corticosteroids, bleomycin, and energy‐based devices. Reported benefits included improved tissue response, enhanced skin texture, and accelerated wound recovery. Key barriers to PN adoption were product cost (66.7%), limited awareness of its role in scar management (52.6%), and injection discomfort (25.2%).

**Conclusion:**

PN injections are commonly incorporated into scar prevention and management among KAAD dermatologists participating in this survey, particularly for acne‐related, surgical, and traumatic scars. These findings provide real‐world insight into current practice patterns and are hypothesis‐generating. Prospective studies are needed to define optimal treatment protocols, including dosing, treatment intervals, and combination strategies.

**Abbreviations**
5‐FU: 5‐fluorouracil
CaHA: Calcium hydroxyapatite
EBD: Energy‐based device
HA: Hyaluronic acid
KAAD: Korean Society for Anti‐Aging Dermatology
LLLT: Low‐level laser therapy
PCL: Polycaprolactone
PDO: Polydioxanone
PDRN: Polydeoxyribonucleotide
PLA: Poly‐L‐lactic acid
PMMA: Polymethylmethacrylate
PN: Polynucleotide
PRP: Platelet‐rich plasma
RF: Radiofrequency

## 1 Introduction

Scarring is a common dermatologic concern arising from acne, trauma, surgery, burns, infections, or aesthetic procedures. While many wounds heal with minimal scarring and some gradually improve over time, others persist and may lead to long‐term cosmetic disfigurement, functional impairment, or psychological distress. Effective scar management is therefore an important goal in both medical and aesthetic dermatology.

Treatment strategies vary according to scar type—atrophic, hypertrophic, or keloidal—as well as the timing of intervention. Preventive measures implemented during early wound healing can substantially reduce long‐term scar burden, whereas established scars often require multimodal corrective approaches. Accordingly, early modulation of the wound‐healing process plays a critical role in optimizing clinical outcomes.

Polynucleotide (PN)–based injectables have gained increasing attention for scar prevention and remodeling. PN preparations used in dermatologic practice are typically derived from salmonid DNA sources such as salmon or trout and have been reported to support wound healing through modulation of the local tissue microenvironment, activation of fibroblasts, and promotion of collagen synthesis [1, 2, 3]. Mechanistically, PN may influence several components of the wound‐healing cascade. Experimental studies suggest that nucleotide derivatives activate purinergic receptors, including P2Y2, which contribute to fibroblast migration, angiogenesis, and extracellular matrix remodeling during tissue repair [1, 3, 4]. In addition, the hydrophilic and viscoelastic properties of PN may help maintain dermal hydration and provide a transient scaffold‐like microenvironment that supports cellular activity within healing tissue [5].

Clinical experience in Korea has reported improvements in skin texture, erythema, and dermal quality following PN treatment, supporting its potential role as a modulatory adjunct in scar management [6, 7, 8, 9]. However, despite increasing clinical use—particularly within Korean aesthetic dermatology—real‐world evidence describing how PN is applied across scar types, treatment stages, and combination strategies remains limited. Most existing publications focus on skin rejuvenation or small case series rather than systematic assessments of routine clinical practice.

To address this gap, we conducted a nationwide survey among dermatologists belonging to the Korean Society for Anti‐Aging Dermatology (KAAD) to characterize real‐world use of PN in scar prevention and management. By examining treatment indications, injection techniques, and combination strategies across different scar types, this study provides a comprehensive overview of current clinical practice and offers real‐world insights that may inform future research and protocol development in PN‐based scar management.

## 2 Materials and Methods

This study was designed as a nationwide, descriptive, real‐world survey to characterize contemporary clinical practices related to PN use in scar prevention and management. The questionnaire assessed the clinical use of PN formulations in general dermatologic practice. In Korea, several PN‐based injectable products are available, and the survey aimed to capture overall clinician experience with PN use in scar prevention and management rather than to evaluate a specific formulation. The study was intended to document usage patterns and clinician perceptions rather than assess comparative efficacy; therefore, inferential statistical analyses and subgroup comparisons were not prespecified.

### 2.1 Study Population

The survey targeted board‐certified members of KAAD, a professional society primarily composed of dermatologists engaged in aesthetic and regenerative dermatology, where PN use is most prevalent in Korea. Participation was voluntary and anonymous.

Eligible respondents were required to have experience with at least five clinical cases involving PN injections. Only respondents who completed all core items related to PN use were included in the analysis.

The survey was distributed to the entire KAAD membership. KAAD currently includes approximately 1 200 dermatologists in Korea. Based on the 501 completed responses, the survey represents a substantial proportion of clinicians within this professional community. For context, Korea currently has approximately 2 900–3 000 board‐certified dermatologists nationwide. Accordingly, the findings reflect the practices of KAAD dermatologists participating in the survey and may not be generalizable to all dermatologists practicing in Korea.

### 2.2 Questionnaire

The questionnaire was developed through expert consensus and reviewed by senior KAAD advisors for clinical relevance and clarity. It included items on respondent demographics and clinical experience, indications for PN use (including scar prevention, atrophic scars, and hypertrophic scars), injection techniques and treatment frequency, combination strategies with injectables and energy‐based devices (EBDs), perceived clinical utility, and barriers to adoption.

The survey consisted of multiple‐choice, matrix, and ranking items, and multiple responses were permitted where applicable. The full questionnaire is provided in Supporting Information 1. Formal psychometric validation, pre‐testing, or pilot testing was not performed.

### 2.3 Data Collection

Data were collected electronically via a secure web‐based platform between August 18 and August 31, 2024. Invitations were distributed to KAAD members by email, and additional participation was encouraged during the KAAD summer meeting. Only respondents who completed all core PN‐related items were included in the final analysis.

### 2.4 Statistical Analysis

Responses were summarized using descriptive statistics. Frequencies and percentages were calculated for categorical variables, including PN usage status, scar‐related indications, injection methods, and combination practices. Data were processed using Microsoft Excel.

### 2.5 Ethics

This study was approved by the Institutional Review Board (IRB). Electronic informed consent was obtained from all participants, and the study was conducted in accordance with the Declaration of Helsinki.

## 3 Results

### 3.1 Respondent Characteristics and Clinical Resources (*n* = 501)

A total of 501 board‐certified dermatologists completed the survey. Because multiple responses were permitted for several survey items, percentages may not sum to 100%. Among respondents, 62.6% reported more than five years of clinical experience after board certification. Most participants indicated access to a broad range of injectable agents and EBDs commonly used in scar management.

Available injectable modalities included botulinum toxin (96.2%), fillers (crosslinked and non‐crosslinked hyaluronic acid [HA], collagen) (91.0%), regenerative agents such as platelet‐rich plasma (PRP) and polydeoxyribonucleotide (PDRN) (86.0%), injectable biostimulators (poly‐L‐lactic acid [PLA], polymethylmethacrylate [PMMA], polydioxanone [PDO], polycaprolactone [PCL], calcium hydroxyapatite [CaHA]) (85.2%), and antifibrotic or tissue‐remodeling agents (e.g., corticosteroids, 5‐fluorouracil [5‐FU], bleomycin) (83.0%). Commonly available devices included pigment lasers (97.4%), vascular lasers (94.6%), ablative fractional lasers (84.6%), invasive radiofrequency (RF) devices (77.6%), and low‐level laser therapy (LLLT) systems (87.0%) (Figure SS1).

### 3.2 Clinical Scenarios for PN Use (*n* = 501)

PN was most frequently considered in cases requiring accelerated wound recovery (80.0%) and for scars located in cosmetically sensitive areas such as the face or neck (64.5%). Other commonly reported indications included wounds under tension or frequent movement (54.1%), large or deep wounds (45.1%), delayed healing associated with systemic conditions such as diabetes (43.1%), keloid‐prone patients (41.5%), scars resulting from procedural complications (18.4%), and pediatric cases (9.6%) (Figure S2).

### 3.3 Barriers to PN Adoption (*n* = 135)

Overall, 366 respondents (73.0%) reported current use of PN for scar management, while 135 reported no prior experience with PN. Among non‐users, the most frequently cited barriers were product cost (66.7%), limited awareness of PN's role in scar management (52.6%), insufficient supporting evidence (37.8%), and injection‐related discomfort (25.2%) (Figure S3).

### 3.4 PN Use for Scar Prevention (*n* = 298)

**FIGURE 1:**
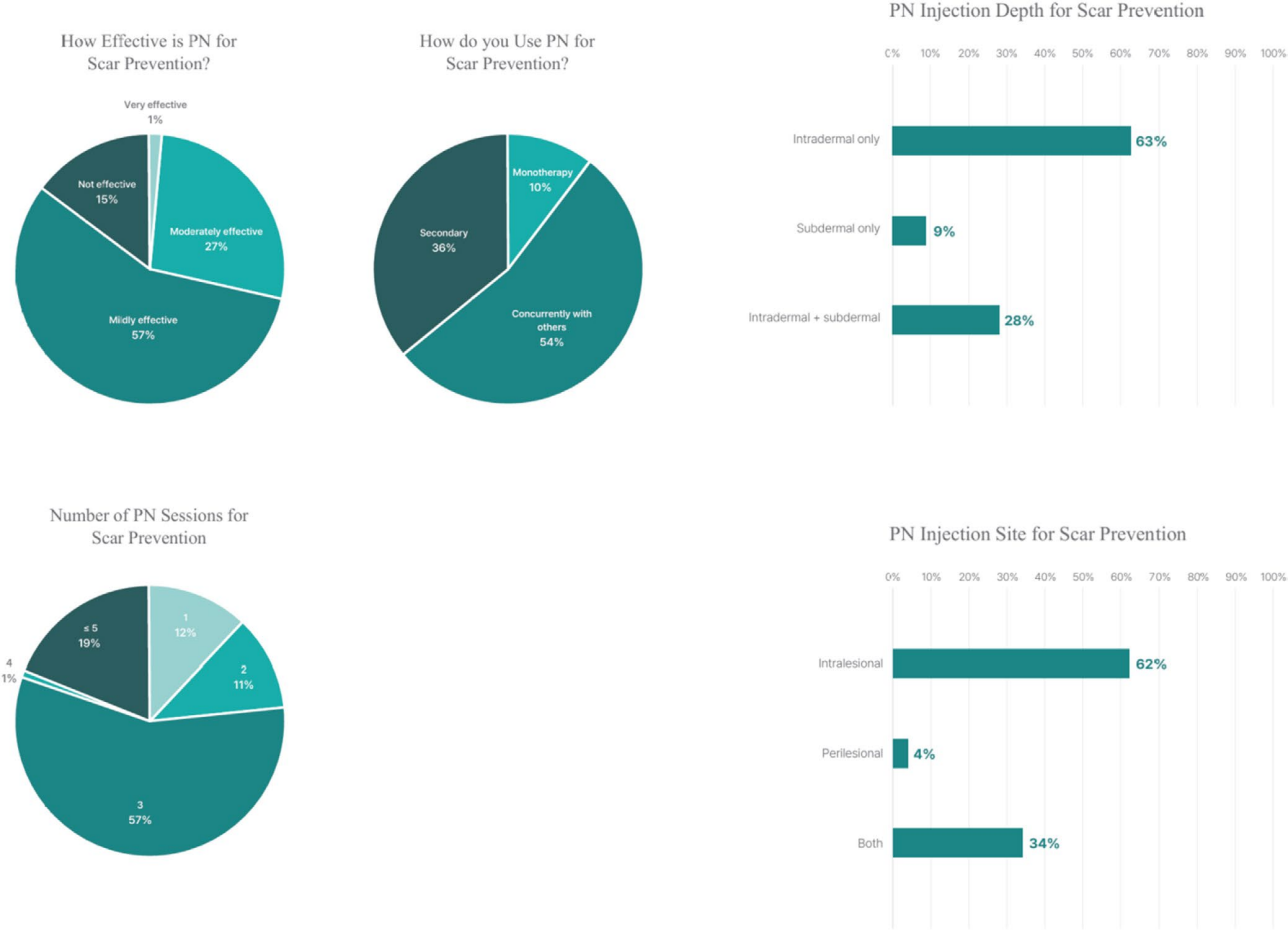
Application of PN injections for scar prevention among PN users, including injection depth, number of sessions, and treatment role (*n* = 298).

Among PN users, 81.4% reported applying PN for scar prevention. Intradermal injection was the most common technique (91.3%), typically delivered directly into the lesion (95.6%) over three treatment sessions (56.7%). PN was most often incorporated into combination treatment protocols (53.7%), followed by secondary use after unsatisfactory outcomes with other treatments (35.9%), whereas monotherapy was less common (10.4%) (Figure 1).

### 3.5 PN Use for Atrophic Scars (*n* = 274)

**FIGURE 2:**
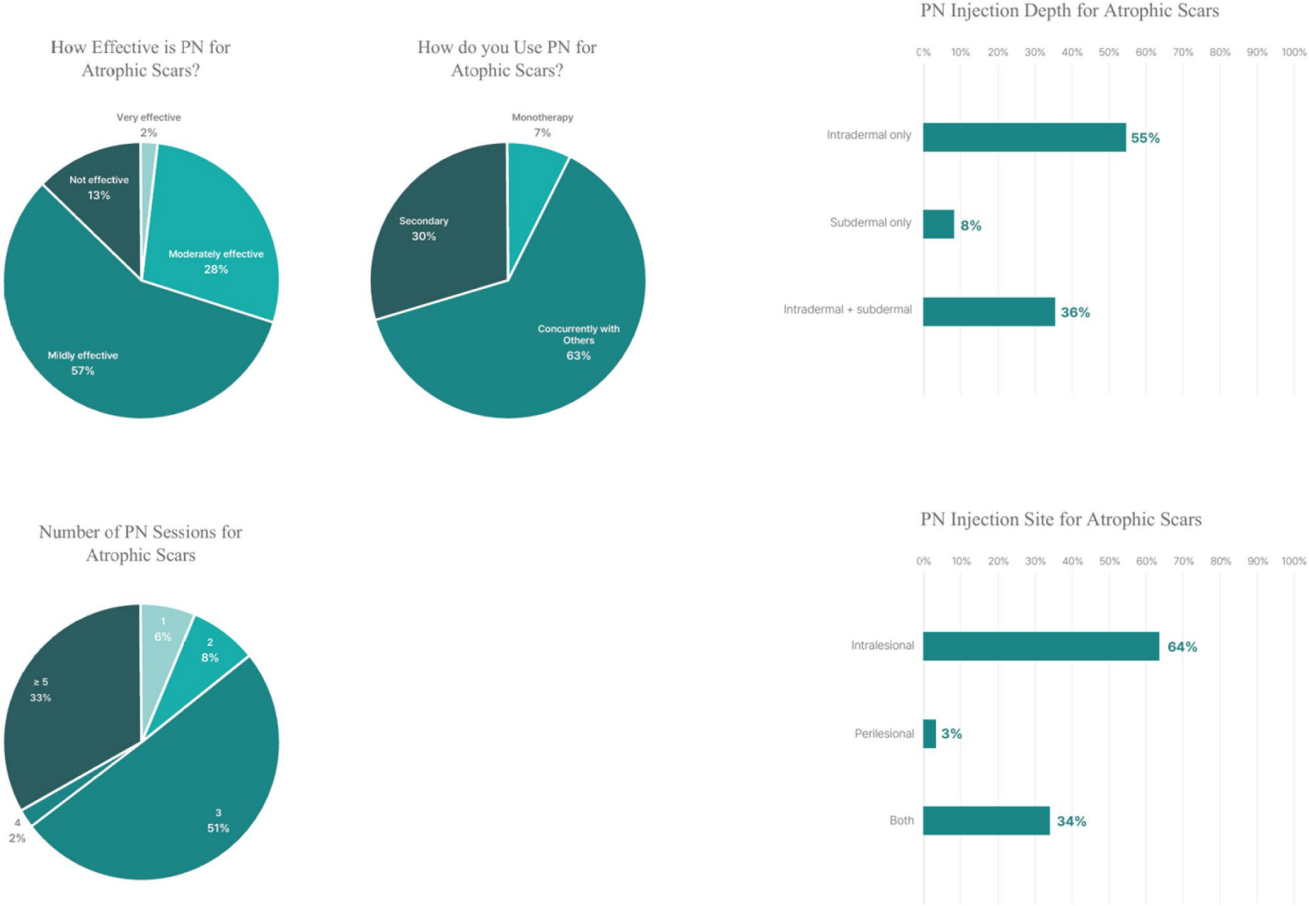
Clinical use patterns of PN injections for atrophic scar improvement (*n* = 274).

PN was used for atrophic scars by 74.9% of respondents. Injections were most frequently administered intradermally (92.0%) and intralesionally (97.4%), commonly over three sessions (50.4%) or five or more sessions (33.2%). PN was most often incorporated into combination treatment strategies (63.1%), followed by secondary use after suboptimal outcomes (29.6%), while monotherapy was reported by 7.3% of respondents (Figure 2).

### 3.6 PN Use for Hypertrophic Scars (*n* = 37)

Use of PN for hypertrophic scars was reported by 10.1% of respondents. PN injections were most commonly administered intradermally (83.8%) and directly into lesions (94.6%), most frequently over three treatment sessions (40.5%). In most cases, PN was combined with other modalities, including antifibrotic agents (56.8%) (Figure S4).

### 3.7 Perceived Rationale Among PN Users (*n* = 366) and Overall Clinical Utility Perceptions (*n* = 501)

**FIGURE 3:**
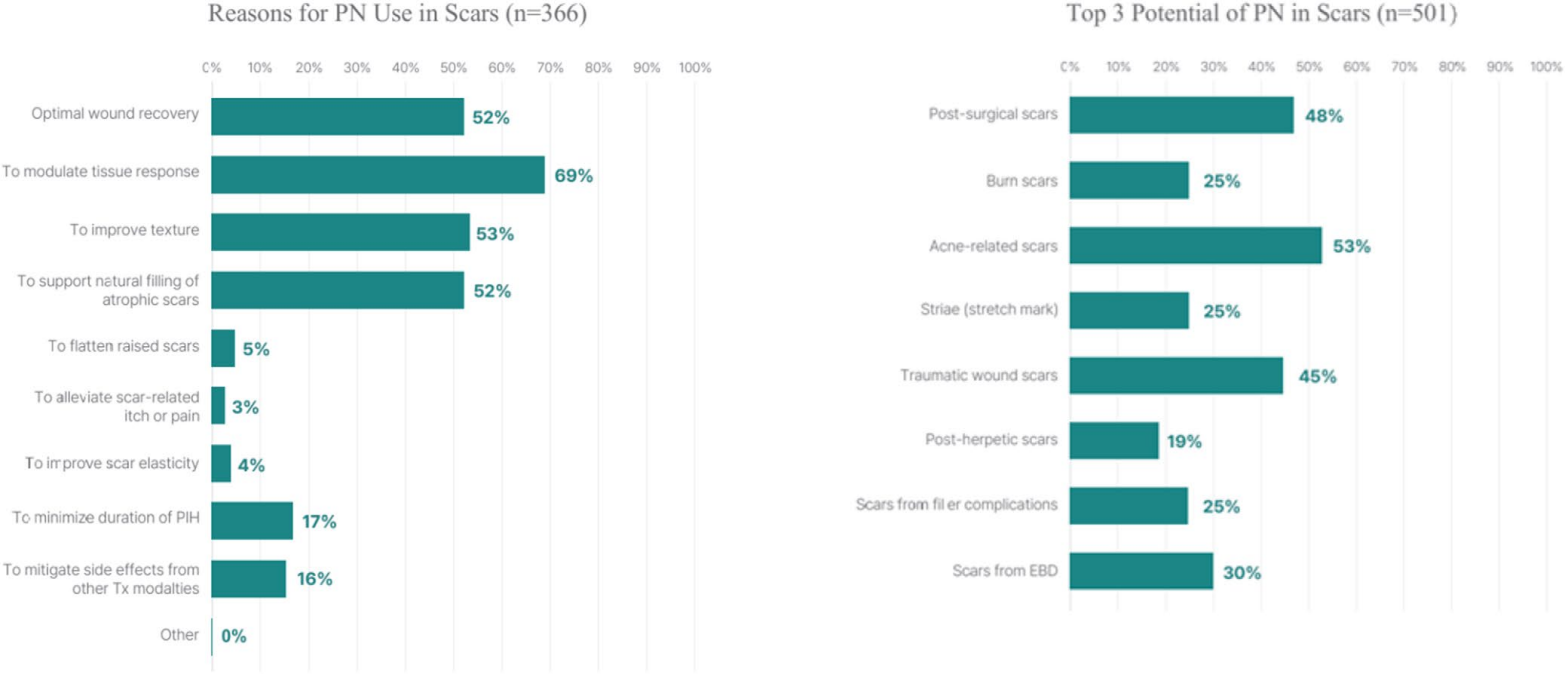
Perceived Rationale Among PN Users (*n* = 366) and Overall Clinical Utility Perceptions (*n* = 501) of PN injections in scar management.

Respondents reported several perceived clinical benefits of PN use, including reduced erythema and tissue reactivity (68.9%), improved skin texture (53.3%), prevention of excessive scar formation (52.2%), and natural volumization of atrophic scars (52.5%) (Figure 3).

Consistent with these perceptions, PN was considered most beneficial for acne scars (52.7%), post‐surgical scars (47.9%), and traumatic scars (45.3%). Additional reported indications included scars related to energy‐based procedures (29.7%), stretch marks (25.0%), filler‐associated necrosis (25.3%), burns or radiation (24.6%), and post–herpes zoster scars (19.2%). (Figure 3). These findings reflect clinician‐reported perceptions rather than patient‐reported outcomes, which were not evaluated in this survey.

### 3.8 PN Application in Acne (*n* = 193), surgical (*n* = 240), and Traumatic (*n* = 227) Scars

PN was widely applied across acne‐related, surgical, and traumatic scars, particularly for preventive and early‐intervention settings.

**FIGURE 4:**
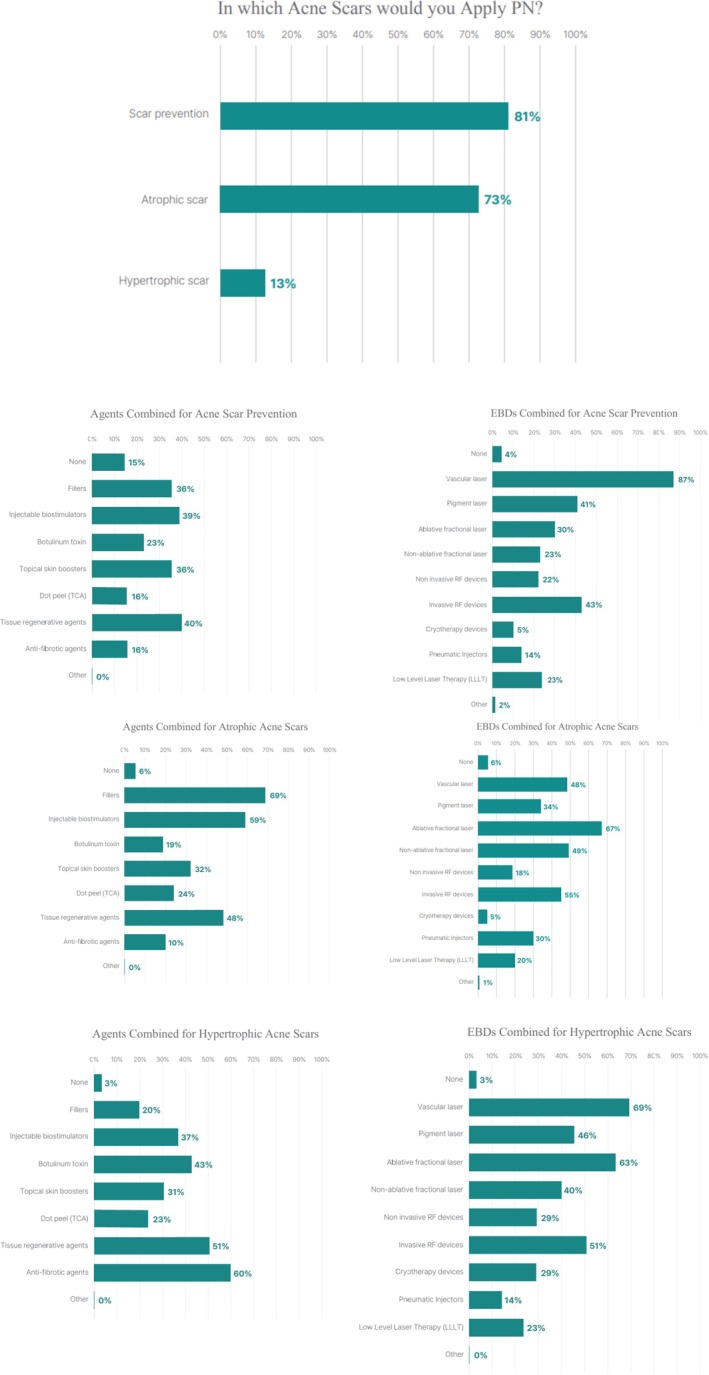
Application of PN injections for acne‐related scars across preventive and corrective settings (*n* = 193).

For acne‐related scarring, PN was most commonly used for post‐acne marks—including postinflammatory erythema and postinflammatory hyperpigmentation (81.1%)—and atrophic acne scars (73.1%), with limited use for hypertrophic acne scars (13.3%). Preventive applications were frequently combined with regenerative agents (47.2%), topical skin boosters (44.9%), and vascular lasers (84.6%). In atrophic acne scars, PN was often paired with fillers (75.1%), biostimulators (59.6%), and regenerative agents (48.7%), whereas hypertrophic acne scars were more commonly treated with antifibrotic agents (63.3%) (Figure 4).

**FIGURE 5:**
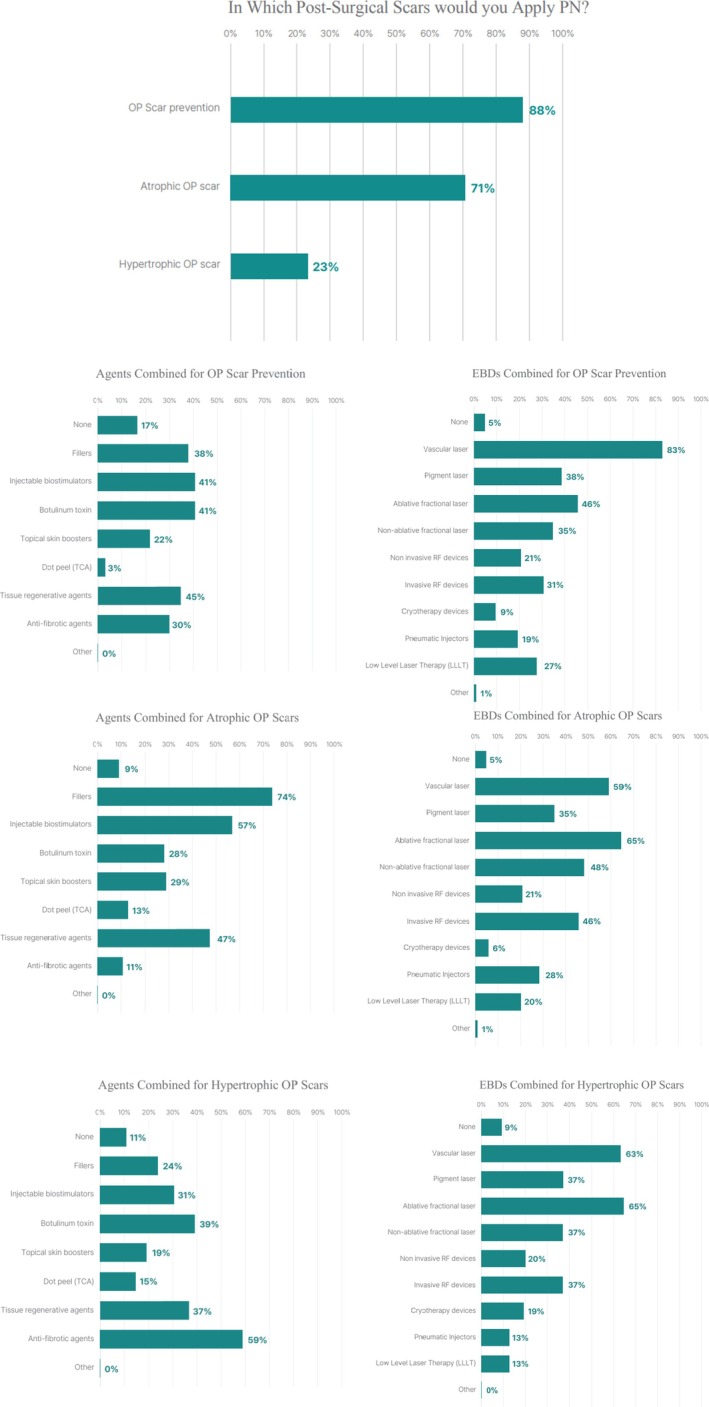
Use of PN injections in surgical (operative, OP) scar prevention and treatment (*n* = 240).

In surgical scars, 88.3% of respondents reported perioperative PN use—defined as administration during surgery or within two weeks after the operation—to reduce postoperative erythema or raised scarring. PN was also used for atrophic (70.8%) and hypertrophic (22.5%) surgical scars. Preventive strategies frequently combined PN with regenerative agents (45.3%), botulinum toxin (40.6%), and vascular lasers (83.5%). For established hypertrophic surgical scars, PN was most often combined with antifibrotic agents (59.3%) and botulinum toxin (38.9%), often alongside laser or RF devices (Figure 5).

**FIGURE 6:**
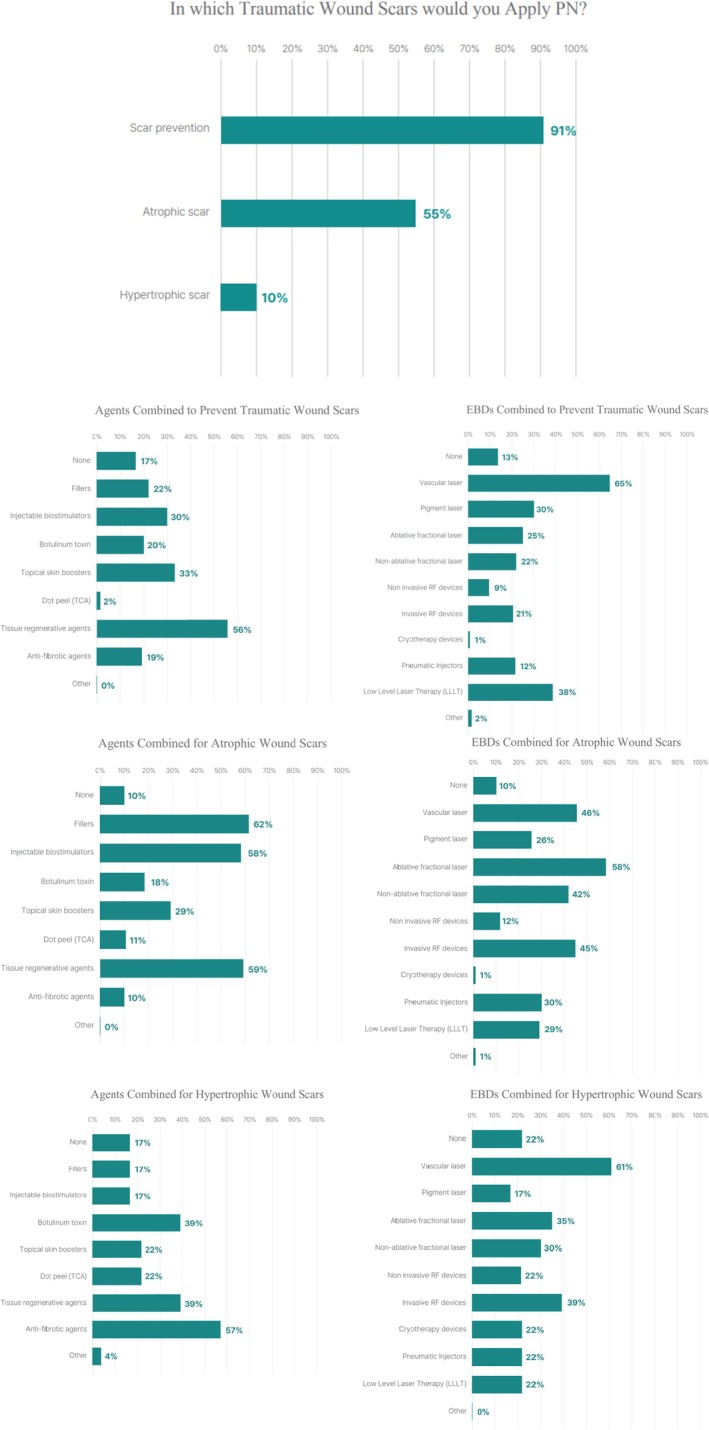
Application of PN Injections for traumatic wound scars (*n* = 227).

For traumatic scars, PN was widely used in early phases to support wound recovery and reduce erythema or pigmentation. Overall, 90.7% of respondents used PN for scar prevention, 55.1% for atrophic scars, and 10.1% for hypertrophic scars. Preventive applications were frequently combined with regenerative agents (55.8%), topical skin boosters (33.5%), vascular lasers (65.0%), LLLT (38.3%), and pigment lasers (30.1%). For established atrophic traumatic scars, PN was often combined with fillers (62.4%), biostimulators (58.4%), and regenerative agents (59.2%), frequently alongside ablative fractional lasers (58.4%), vascular lasers (45.6%), and RF devices (44.8%) (Figure 6).

## 4 Discussion

This nationwide survey provides real‐world insight into current patterns of PN injection use in scar prevention and management among KAAD dermatologists. By design, the study aimed to characterize contemporary practice patterns and clinician perceptions rather than evaluate comparative efficacy or test predefined hypotheses. Accordingly, the findings should be interpreted as hypothesis‐generating and informative for clinical practice.

A substantial proportion of respondents (73%) reported using PN in scar management, most commonly for scar prevention and the treatment of atrophic scars. This usage pattern reflects the perceived role of PN as a modulatory agent in wound healing and is consistent with previously reported PN‐associated effects on cellular responses, fibroblast activity, and extracellular matrix organization—processes central to the wound‐healing cascade [1, 2, 3, 10, 11, 12, 13, 14]. The frequent preventive use of PN, particularly in surgical, traumatic, and post‐acne settings, suggests growing clinical interest in early intervention strategies aimed at reducing unfavorable scar formation.

The study population consisted of KAAD members, a group highly engaged in aesthetic and regenerative dermatology, including injectable therapies and scar management. Because PN use in Korea is largely concentrated within this clinical context, KAAD dermatologists were considered an appropriate population to capture real‐world PN utilization patterns. However, this focus may limit generalizability to dermatologists practicing in settings with less emphasis on aesthetic or regenerative procedures.

In clinical practice, PN was most commonly administered intradermally over multiple treatment sessions, suggesting a degree of informal consensus regarding technique. Nevertheless, variability in injection depth and approach—particularly in hypertrophic scars—indicates that clinicians adapt treatment strategies based on scar morphology and mechanical tension. Respondents represented a range of clinical experience levels, with more than 60% reporting over five years of post‐certification practice. Although clinical experience was captured descriptively, the study was not designed to evaluate practice differences according to experience or case volume.

Combination therapy emerged as a central feature of PN use across scar types. PN was rarely used as monotherapy and was more often incorporated into multimodal protocols involving botulinum toxin, fillers, biostimulators, antifibrotic agents, and EBDs. Recent clinical evidence further supports the potential role of PN‐based combination therapies in acne scar management. For example, a prospective multicenter real‐world study by Lim et al. [15]. reported improvements in dermal quality and scar appearance using a combination of high‐purification PN technology and hyaluronic acid for moderate‐to‐severe atrophic acne scars. These findings align with the treatment patterns observed in our survey, where PN was frequently combined with fillers and other regenerative approaches. Such combinations likely reflect complementary mechanisms rather than standardized treatment algorithms [8, 16, 17, 18, 19]. Importantly, our survey did not assess treatment sequencing, intervals, or protocol rationale, which limits direct clinical translation and highlights the need for prospective studies evaluating standardized PN‐based treatment protocols. A conceptual framework summarizing clinician‐reported PN application across scar maturation stages is provided (Figure S5).

Respondents also reported perceived clinical benefits of PN use, including reduced erythema, improved skin texture, prevention of excessive scar formation, and natural volumization of atrophic scars. PN was considered particularly beneficial for acne, surgical, traumatic, and post–herpes zoster scars. While these perceptions are consistent with previous clinical reports [4, 5, 20, 21, 22, 23], they represent clinician‐reported impressions rather than objective outcome measures. In addition, scar classifications were based on clinician interpretation without standardized diagnostic criteria or photographic validation, which may introduce variability.

Several barriers to PN adoption were identified, most notably product cost, limited awareness of its role in scar management, and insufficient supporting evidence. These findings highlight ongoing challenges in the broader integration of regenerative therapies into routine clinical practice. Injection discomfort was also reported as a barrier, suggesting that formulation or technique modifications may improve patient acceptance, particularly in preventive or aesthetic settings.

The absence of inferential statistics and subgroup comparisons reflects the descriptive design of this study. In addition, participation was voluntary, and respondents may represent clinicians with greater interest or experience in PN‐based treatments, which may introduce response bias. Nevertheless, future investigations could benefit from stratified analyses (e.g., PN users vs. non‐users, clinical experience level, or practice setting) and the inclusion of confidence intervals to enhance interpretability. Similarly, formal validation of survey instruments would improve internal consistency and reproducibility.

This study reflects clinician experience with PN‐based injectable formulations used in routine dermatologic practice in Korea. Although certain PN products are widely used in the Korean aesthetic market, the survey was designed to capture overall PN utilization patterns rather than evaluate specific formulations.

Finally, while most existing PN literature focuses on skin rejuvenation or small clinical series, this survey provides a large‐scale real‐world perspective on PN use in scar prevention and management. International comparative data remain limited, and future multinational surveys or collaborative registries may help clarify regional differences in PN adoption, treatment techniques, and clinical outcomes.

## 5 Conclusion

This survey demonstrates that PN injections are commonly incorporated into scar prevention and management among KAAD dermatologists participating in this study. PN is used in both preventive and corrective settings and is frequently integrated into multimodal treatment strategies, reflecting its perceived clinical utility in real‐world practice.

Although these findings describe current practice patterns rather than establish comparative efficacy, they provide a useful foundation for future research. Prospective controlled studies, standardized treatment protocols, and long‐term outcome data are needed to better define the optimal role of PN—including appropriate dosing, treatment intervals, and combination strategies—within evidence‐based scar management.

## Funding

This work was supported by PharmaResearch.

## Ethics Statement

The study was approved by the Institutional Review Board (IRB) (IRB No. OC25QISI0153). Electronic informed consent was obtained, and the study adhered to the principles of the Declaration of Helsinki.

## Consent

Informed consent was obtained electronically from all participants prior to participation in the survey.

## Conflicts of Interest

Nark Kyoung Rho is on the advisory board of PharmaResearch Co. Ltd.

## Supporting information


**Figure S1:** Respondent characteristics and available clinical resources (*n* = 501).
**Figure S2:** Clinical scenarios in which PN use was considered (*n* = 501).
**Figure S3:** Barriers to PN adoption in clinical practice (*n* = 135).
**Figure S4:** Use of PN injections for hypertrophic scars (*n* = 37).
**Figure S5:** Conceptual recommendation framework for PN application according to scar maturation stage and overall risk of pathologic scarring, based on aggregated clinician responses. This figure reflects descriptive survey responses and does not represent statistically derived recommendations.

**Data S1:** jocd70887‐sup‐0002‐Supinfo1_Supplementary.docx.

## Data Availability

The data that support the findings of this study are available from the corresponding author upon reasonable request. The survey dataset contains anonymized responses from participating dermatologists and is not publicly available due to privacy and institutional restrictions.

## References

[1] M. P. Rathbone , P. J. Middlemiss , J. W. Gysbers , S. DeForge , P. Costello , and R. F. Del Maestro , “Purine Nucleosides and Nucleotides Stimulate Proliferation of a Wide Range of Cell Types,” Vitro Cell Development Biology 28a, no. 7–8 (1992): 529–536, 10.1007/bf02634137. 1522046

[2] R. Lemmens , L. Vanduffel , H. Teuchy , and O. Culic , “Regulation of Proliferation of LLC‐MK2 Cells by Nucleosides and Nucleotides: The Role of Ecto‐Enzymes,” Biochemical Journal 316 (1996): 551–557, 10.1042/bj3160551. 8687400 PMC1217384

[3] H. Jin , J. Seo , S. Y. Eun , et al., “P2Y2 R Activation by Nucleotides Promotes Skin Wound‐Healing Process,” Experimental Dermatology 23, no. 7 (2014): 480–485, 10.1111/exd.12440. 24816122

[4] F. Squadrito , A. Bitto , N. Irrera , et al., “Pharmacological Activity and Clinical Use of PDRN,” Frontiers in Pharmacology 8 (2017): 224, 10.3389/fphar.2017.00224. 28491036 PMC5405115

[5] K. W. A. Lee , K. W. L. Chan , A. Lee , et al., “Polynucleotides in Aesthetic Medicine: A Review of Current Practices and Perceived Effectiveness,” International Journal of Molecular Sciences 25, no. 15 (2024): 8224, 10.3390/ijms25158224. 39125793 PMC11311621

[6] Y. J. Lee , H. T. Kim , Y. J. Lee , et al., “Comparison of the Effects of Polynucleotide and Hyaluronic Acid Fillers on Periocular Rejuvenation: A Randomized, Double‐Blind, Split‐Face Trial,” Journal of Dermatological Treatment 33, no. 1 (2022): 254–260, 10.1080/09546634.2020.1748857. 32248707

[7] K. Y. Park , J. Seok , N. K. Rho , B. J. Kim , and M. N. Kim , “Long‐Chain Polynucleotide Filler for Skin Rejuvenation: Efficacy and Complications in Five Patients,” Dermatologic Therapy 29, no. 1 (2016): 37–40, 10.1111/dth.12299. 26814448

[8] N. K. Rho , K. H. Han , M. Cho , and H. S. Kim , “A Survey on the Cosmetic Use of Injectable Polynucleotide: The Pattern of Practice Among Korean Dermatologists,” Journal of Cosmetic Dermatology 23, no. 4 (2024): 1243–1252, 10.1111/jocd.16125. 38093498

[9] D. Lee , M. J. Kim , H. J. Park , et al., “Current Practices and Perceived Effectiveness of Polynucleotides for Treatment of Facial Erythema by Cosmetic Physicians,” Skin Research and Technology 29, no. 9 (2023): e13466, 10.1111/srt.13466. 37753681 PMC10485387

[10] J. H. Kim , J. J. Jeong , Y. I. Lee , et al., “Preventive Effect of Polynucleotide on Post‐Thyroidectomy Scars: A Randomized, Double‐Blinded, Controlled Trial,” Lasers in Surgery and Medicine 50, no. 7 (2018): 755–762, 10.1002/lsm.22812. 29574803

[11] N.‐K. Rho and S. T. Chung , “Treatment of a Full‐Thickness Laceration Scar on the Forehead Using a 1,064‐Nm Fractional Picosecond Laser and Polynucleotide Gel Injection,” Medical Lasers 7, no. 2 (2018): 88–90, 10.25289/ML.2018.7.2.88.

[12] M. J. Kim , H. J. Park , S. M. Oh , and K. H. Yi , “Polynucleotide Injection Treatment for Iatrogenic Fat Atrophy in Two Patients: Potential for Safe Volumization in Aesthetic Medicine,” Skin Research and Technology 29, no. 8 (2023): e13439, 10.1111/srt.13439. 37632185 PMC10423761

[13] A. Araco and F. Araco , “Preliminary Prospective and Randomized Study of Highly Purified Polynucleotide vs Placebo in Treatment of Moderate to Severe Acne Scars,” Aesthetic Surgery Journal 41, no. 7 (2021): Np866–np874, 10.1093/asj/sjab125. 33755110

[14] I. P. D. L. C. Palmieri , “Striae Distensae and an Innovative Intradermal Medical Device Based on PN HPT, Hyaluronic Acid, and Mannitol. A Real‐World Insight,” J Case Rep Med Hist 4, no. 4 (2024): 1–9, 10.54289/JCRMH2400116.

[15] T. S. Lim , C. Ian , N. A. Abdullah , et al., “Prospective Multicentre Real‐World Study of a Bioregenerative Combination Therapy With Polynucleotide High‐Purification Technology (PN HPT) and Hyaluronic Acid for Moderate‐To‐Severe Atrophic Facial Acne Scars,” Cosmetics 13, no. 1 (2026): 1.

[16] Y. S. Kim , E. S. Hong , and H. S. Kim , “Botulinum Toxin in the Field of Dermatology: Novel Indications,” Toxins (Basel) 9, no. 12 (2017): 1–17, 10.3390/toxins9120403. PMC5744123 29258169

[17] S. B. Han , H. Kim , S. H. Cho , J. H. Chung , and H. S. Kim , “Protective Effect of Botulinum Toxin Type A Against Atopic Dermatitis‐Like Skin Lesions in NC/Nga Mice,” Dermatologic Surgery 43, no. Suppl 3 (2017): S312–s321, 10.1097/dss.0000000000001170. 28445195

[18] Y. S. Kim , H. J. Lee , S. H. Cho , J. D. Lee , and H. S. Kim , “Early Postoperative Treatment of Thyroidectomy Scars Using Botulinum Toxin: A Split‐Scar, Double‐Blind Randomized Controlled Trial,” Wound Repair Regen. Sep‐Oct 22, no. 5 (2014): 605–612, 10.1111/wrr.12204. 24898579

[19] S. Kim , Y. R. Woo , S. H. Cho , J. D. Lee , and H. S. Kim , “Clinical Efficacy of 5‐Fluorouracil and Bleomycin in Dermatology,” Journal of Clinical Medicine 13, no. 2 (2024): 335, 10.3390/jcm13020335. 38256469 PMC10816055

[20] M. J. Kim , J. Wan , L. Oksana , et al., “Polynucleotide‐Based Treatments for Various Facial Scars Including Combat Injuries,” Journal of Dermatological Treatment 35, no. 1 (2024): 2426626, 10.1080/09546634.2024.2426626. 39561983

[21] F. Veronesi , D. Dallari , G. Sabbioni , C. Carubbi , L. Martini , and M. Fini , “Polydeoxyribonucleotides (PDRNs) From Skin to Musculoskeletal Tissue Regeneration via Adenosine A(2A) Receptor Involvement,” Journal of Cellular Physiology 232, no. 9 (2017): 2299–2307, 10.1002/jcp.25663. 27791262

[22] O. Muratore , G. Cattarini , S. Gianoglio , et al., “A Human Placental Polydeoxyribonucleotide (PDRN) May Promote the Growth of Human Corneal Fibroblasts and Iris Pigment Epithelial Cells in Primary Culture,” New Microbiologica 26, no. 1 (2003): 13–26. 12578307

[23] D. S. Kim , J. K. Lee , J. H. Kim , et al., “Advanced PLGA Hybrid Scaffold With a Bioactive PDRN/BMP2 Nanocomplex for Angiogenesis and Bone Regeneration Using Human Fetal MSCs,” Science Advances 7, no. 50 (2021): eabj1083, 10.1126/sciadv.abj1083. 34878837 PMC8654289

