# Supplementary material for: Real‐World Use of Polynucleotide Injections in Scar Prevention and Management: A Nationwide Survey of Korean Society for Anti‐Aging Dermatology (KAAD) Dermatologists
Source: J Cosmet Dermatol. 2026 May 5;25:e70887. doi: 10.1111/jocd.70887 (PMC13144716; doi:10.1111/jocd.70887)
Supplement: Supplementary file 1 — Figure S1: Respondent characteristics and available clinical resources (n = 501). Figure S2: Clinical scenarios in which PN use was considered (n = 501). Figure S3: Barriers to PN adoption in clinical practice (n = 135). Figure S4: Use of PN injections for hypertrophic scars (n = 37). Figure S5: Conceptual recommendation framework for PN application according to scar maturation stage and overall risk of pathologic scarring, based on aggregated clinician responses. This figure reflects descriptive survey responses and does not represent statistically derived recommendations. [file JOCD-25-e70887-s002.docx]

**
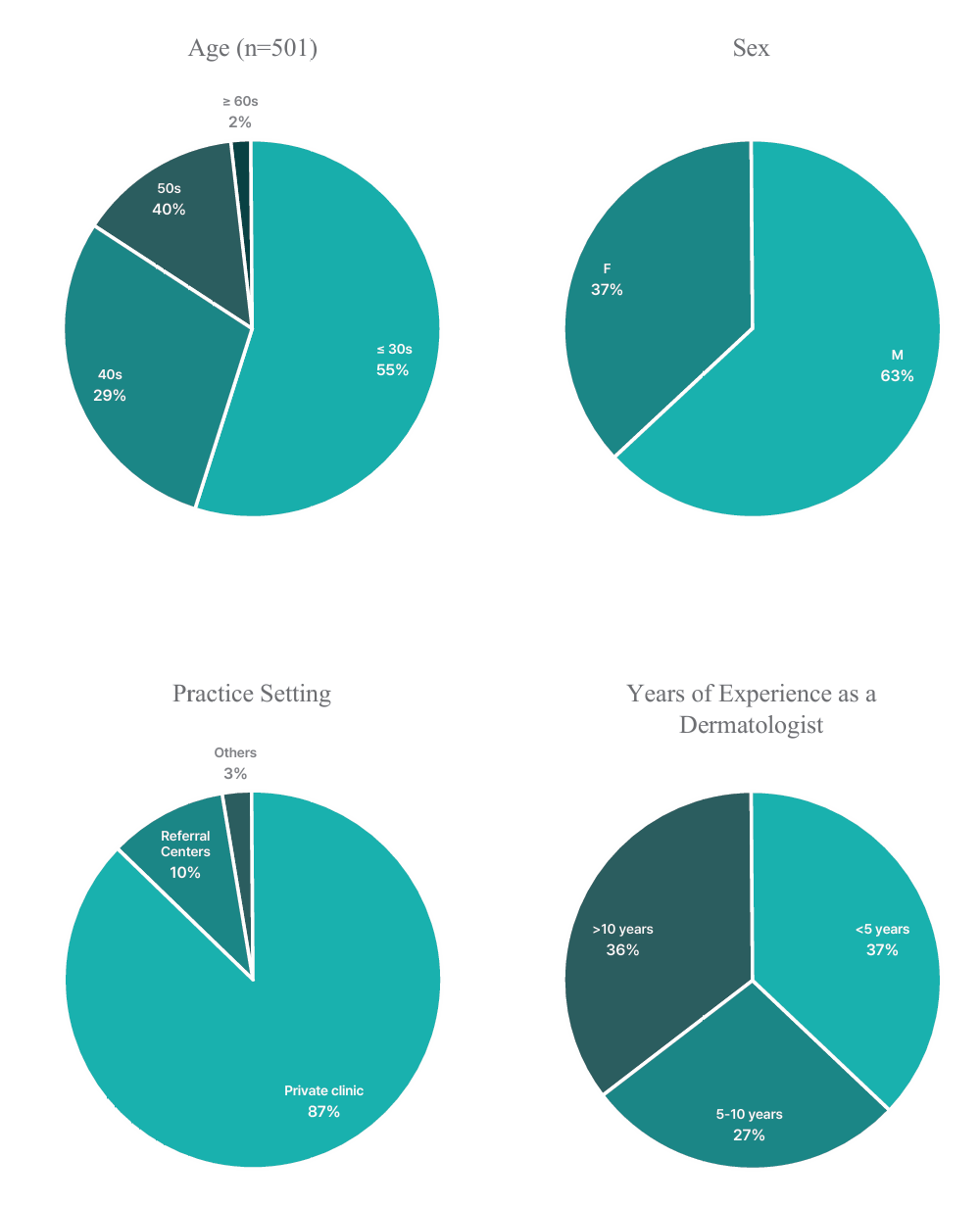
Supplementary Figure 1. Respondent characteristics and available clinical resources (n = 501).**

**
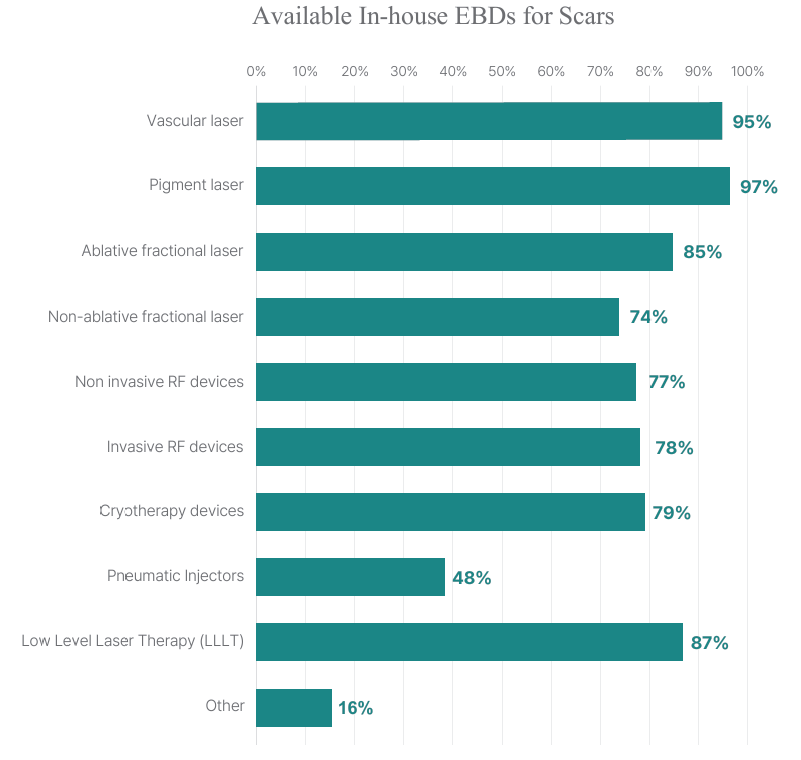

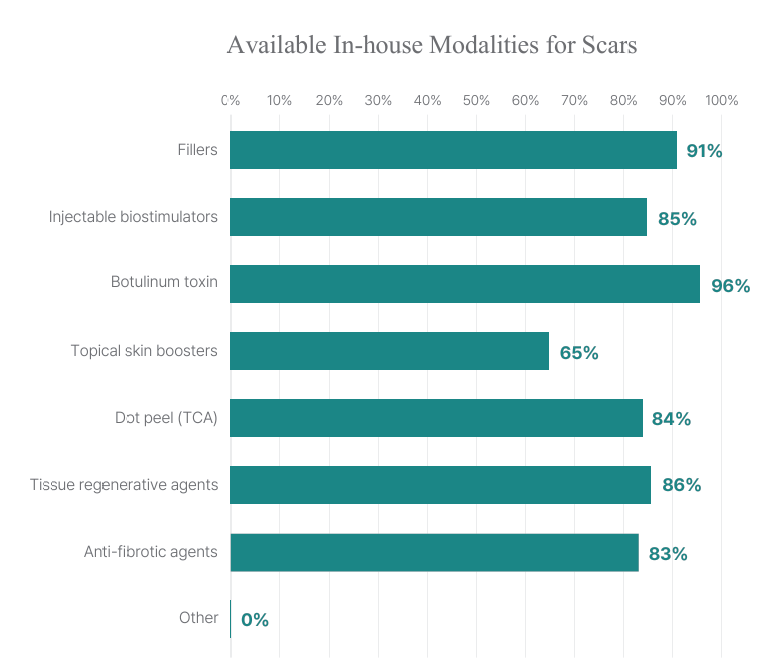
**

**Supplementary Figure 2. Clinical scenarios in which PN use was considered (n = 501).**

**
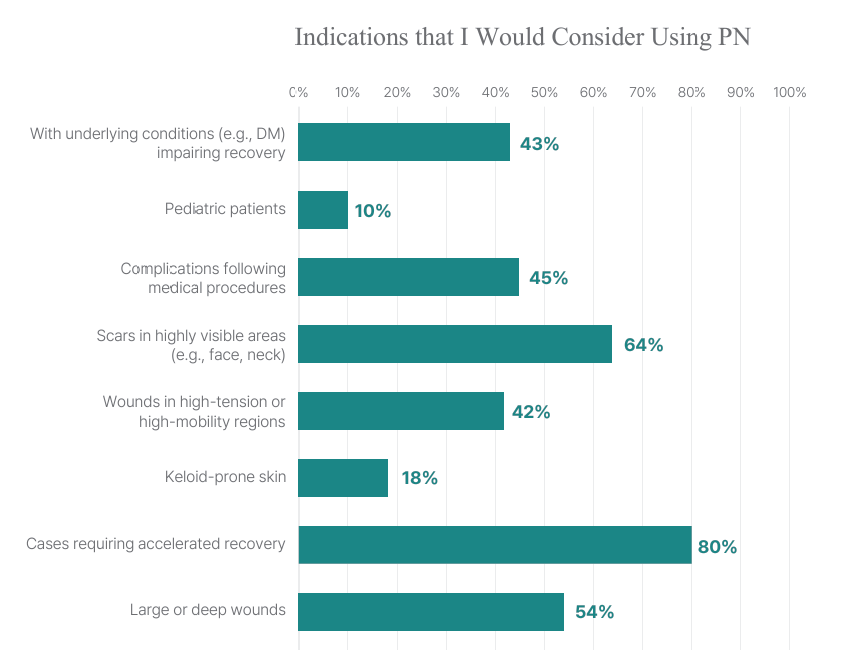
**

**Supplementary Figure 3. Barriers to PN adoption in clinical practice (n = 135).**

**
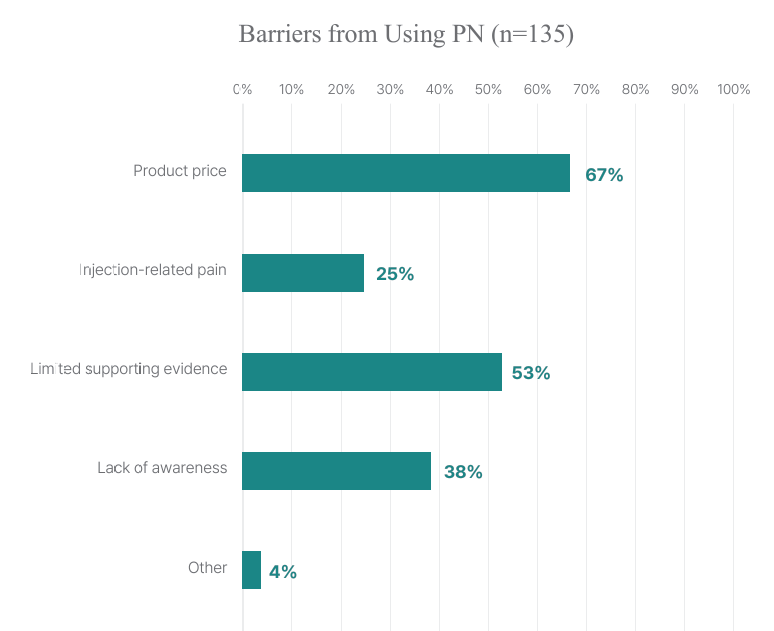
**

**
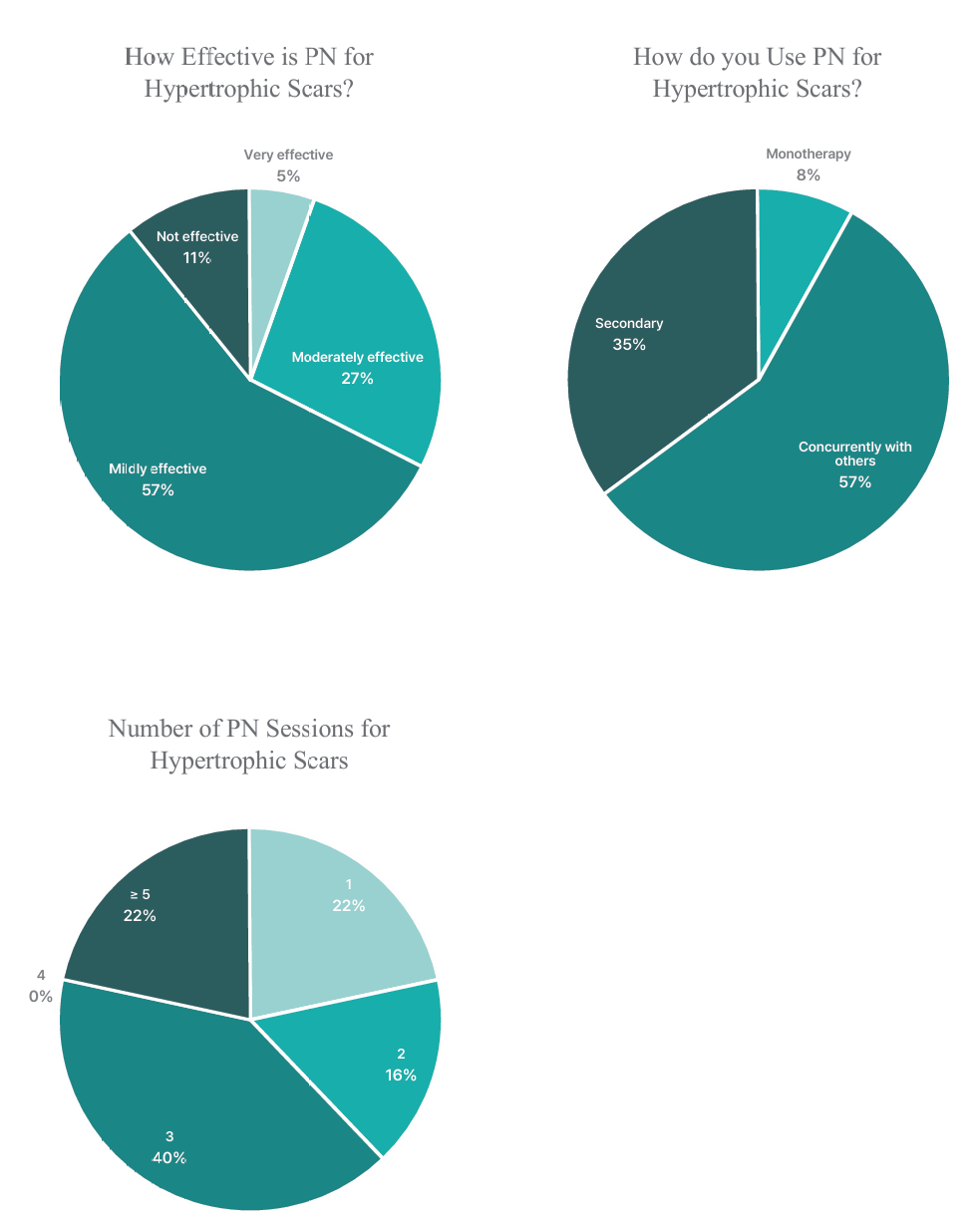
****Supplementary Figure 4. Use of PN injections for hypertrophic scars (n = 37).**

**
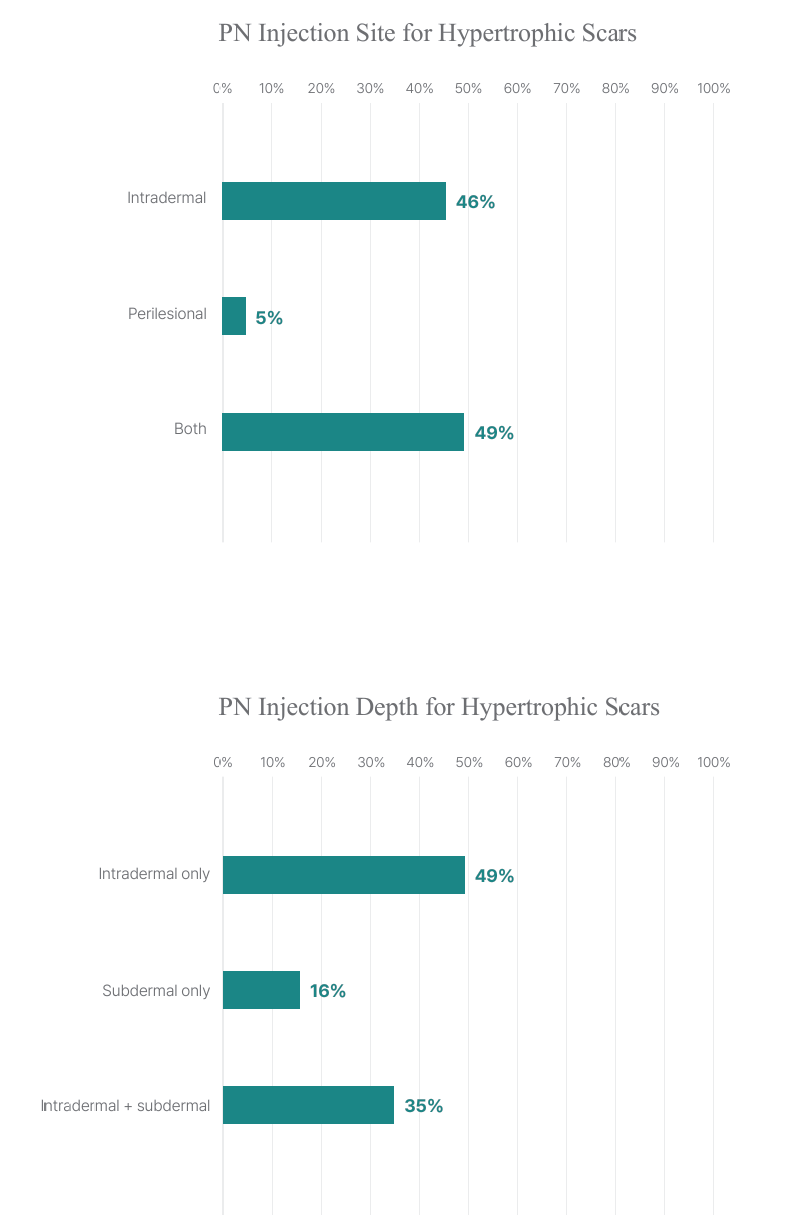
**

**Supplementary Figure 5. Conceptual recommendation framework for PN application according to scar maturation stage and overall risk of pathologic scarring, based on aggregated clinician responses. This figure reflects descriptive survey responses and does not represent statistically derived recommendations.**

**
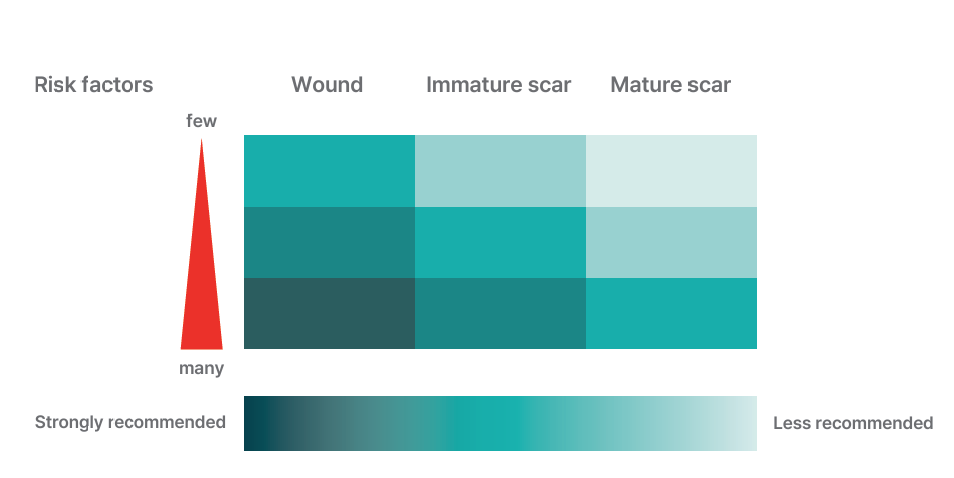
**
