# Supplementary material for: Real‐World Use of Polynucleotide Injections in Scar Prevention and Management: A Nationwide Survey of Korean Society for Anti‐Aging Dermatology (KAAD) Dermatologists
Source: J Cosmet Dermatol. 2026 May 5;25:e70887. doi: 10.1111/jocd.70887 (PMC13144716; doi:10.1111/jocd.70887)
Supplement: Supplementary file 2 — Data S1: jocd70887‐sup‐0002‐Supinfo1_Supplementary.docx. [file JOCD-25-e70887-s001.docx]

**Supplementary Material 1. Full Survey Questionnaire**

**Title:**

Survey on the Use and Potential of Polynucleotide (PN, e.g., REJURAN^®^) in Scar Management

**Purpose:**

To investigate real-world PN usage patterns in scar prevention and management.

To assess clinical perceptions of efficacy, administration methods, and combination approaches.

**Instructions:**

This survey is intended for dermatologists with experience using PN (e.g., REJURAN^®^). Please respond based on your clinical practice.

**Section A. Screening and Respondent Characteristics**

A1. Have you used PN (e.g., REJURAN^®^) in clinical practice?

(Please select “Yes” if you have performed at least five cases, regardless of indication.)

☐ Yes

☐ No → Thank you. You are not eligible to participate in this survey. (Survey ends.)

A2. Please select your age group:

☐ 30s or younger

☐ 40s

☐ 50s

☐ 60s

☐ 70s or older

A3. Please indicate your gender:

☐ Male

☐ Female

A4. Please indicate your current practice setting:

☐ Private dermatology clinic

☐ Hospital-based dermatology department (general or university hospital)

☐ Other: ___________

A5. Please indicate your experience as a board-certified dermatologist:

☐ < 5 years

☐ 5–10 years

☐ > 10 years

**Section B. PN Experience and Consideration for Scar-Related Use**

(To be completed by both PN users and non-users)

B1. Have you ever used PN (e.g., REJURAN^®^) for the management or prevention of scars?

(Scars are defined here as including persistent erythema, pigmentation, textural irregularities, or visible depressions or elevations.)

☐ Yes → Proceed to Section C

☐ No

B2. In which of the following clinical situations would you consider using PN (e.g., REJURAN^®^)?

(Select all that apply)

☐ Patients with systemic conditions (e.g., diabetes) that impair wound recovery

☐ Pediatric patients

☐ Complications or sequelae following medical procedures

☐ Scars in highly visible areas (e.g., face, neck)

☐ Wounds in high-tension or high-mobility regions

☐ Patients with keloid-prone skin

☐ Cases requiring accelerated recovery

☐ Large or deep wounds with high scarring potential

B3. If you have not used PN for scar prevention or treatment, what are your reasons?

(Select all that apply)

☐ Product price

☐ Injection-related discomfort

☐ Limited supporting evidence for scar management

☐ Lack of awareness for PN in scars

☐ Other: ___________

→ Proceed to Section H (skip Section C–G)

**Section C. PN Use in Scars**

(To be completed only by respondents who answered "Yes" to B1)

C1. For which types of scars have you used PN (e.g., REJURAN^®^)?

(Select all that apply)

☐ Scar prevention (e.g., immediately after surgery, ablative laser, or resolving acne lesions with erythema)

☐ Atrophic scars (e.g., acne pits, surgical depressions)

☐ Hypertrophic scars (raised or firm scars)

**Section D. Detailed Practices in PN Use for Scar Prevention**

D1. Based on your experience, how effective is PN (e.g., REJURAN^®^) for scar prevention?

☐ (4) Very effective –Suitable as monotherapy

☐ (3) Moderately effective – Useful as an adjunct to other modalities (e.g., triamcinolone, bleomycin, lasers, botulinum toxin, subcision, TCA)

☐ (2) Mildly effective – Considered for patients seeking optimal results regardless of cost

☐ (1) Not effective – No plans to use

D2. Typical injection depths for PN in scar prevention:

(Select all that apply)

☐ Intradermal

☐ Subdermal

D3. Typical number of PN sessions for scar prevention:

☐ 1

☐ 2

☐ 3

☐ 4

☐ ≥ 5

D4. Typical injection sites for scar prevention:

(Select all that apply)

☐ Into the lesion (intralesional)

☐ Around the lesion (perilesional)

D5. PN use strategy in scar prevention:

☐ Used as a monotherapy (first-line)

☐ Used concurrently with other modalities (e.g., lasers, subcision, TCA, bleomycin, triamcinolone, botulinum toxin)

☐ Added later as a secondary approach when prior efficacy was limited or to reduce side effects (e.g., steroid-induced atrophy or redness)

**Section E. PN Use for Atrophic Scar Treatment**

E1. Based on your experience, how effective is PN (e.g., REJURAN^®^) for atrophic scars?

☐ (4) Very effective – Suitable as monotherapy

☐ (3) Moderately effective – Useful as an adjunct to other modalities (e.g., triamcinolone, bleomycin, lasers, botulinum toxin, subcision, TCA)

☐ (2) Mildly effective – Considered for patients seeking optimal results regardless of cost

☐ (1) Not effective – No plans to use

E2. Typical injection depths for PN in atopic scars:

(Select all that apply)

☐ Intradermal

☐ Subdermal

E3. Typical number of PN sessions for atrophic scars:

☐ 1

☐ 2

☐ 3

☐ 4

☐ ≥ 5

E4. Typical injection sites for atrophic scars:

(Select all that apply)

☐ Into the lesion (intralesional)

☐ Around the lesion (perilesional)

E5. PN use strategy in atrophic scars:

☐ Used as monotherapy (first-line)

☐ Used concurrently with other modalities (e.g., lasers, subcision, TCA, bleomycin, triamcinolone, botulinum toxin)

☐ Added later as a secondary approach when prior efficacy was limited or to reduce side effects (e.g., steroid-induced atrophy or redness)

**Section F. PN Use for Hypertrophic Scar Treatment**

F1. Based on your experience, how effective is PN (e.g., REJURAN^®^) for hypertrophic scars?

☐ (4) Very effective –Suitable as monotherapy

☐ (3) Moderately effective – Useful as an adjunct to other modalities (e.g., triamcinolone, bleomycin, lasers, botulinum toxin, subcision, TCA)

☐ (2) Mildly effective – Considered for patients seeking the optimal results regardless of cost

☐ (1) Not effective – No plans to use

F2. Typical injection depths for PN in hypertrophic scars:

(Select all that apply)

☐ Intradermal

☐ Subdermal

F3. Typical number of PN sessions for hypertrophic scars:

☐ 1

☐ 2

☐ 3

☐ 4

☐ ≥ 5

F4. Typical injection sites for hypertrophic scars:

(Select all that apply)

☐ Into the lesion (intralesional)

☐ Around the lesion (perilesional)

F5. PN use strategy in hypertrophic scars:

☐ Used as monotherapy (first-line)

☐ Used concurrently with other modalities (e.g., lasers, subcision, TCA, bleomycin, triamcinolone, botulinum toxin)

☐ Added later as a secondary approach when prior efficacy was limited or to reduce side effects (e.g., steroid-induced atrophy or redness)

**Section G. Reasons for Using PN (e.g., REJURAN^®^) in Scars**

(Please select your top 3 reasons, in order of importance)

☐ To promote optimal wound recovery and prevent significant depression or elevation of scars

☐ To modulate tissue response and reduce the duration of redness

☐ To improve the texture and surface smoothness

☐ To support natural remodeling and filling of atrophic scars

☐ To soften and flatten raised scars

☐ To alleviate scar-related symptoms such as itching or tenderness

☐ To enhance elasticity and reduce rigidity

☐ To minimize pigmentation or shorten the duration of post-procedural discoloration

☐ To mitigate side effects from other modalities (e.g., steroid-induced atrophy, redness)

☐ Other: ___________

**Section H. Perceived Potential of PN for Various Scar Indications**

(To be answered by all respondents, regardless of PN use)

H1. In which of the following clinical situations do you believe PN (e.g., REJURAN^®^) injections have high potential?

(Select up to 3 options)

☐ Post-surgical scar prevention/management

☐ Burn- or post-radiation scar prevention/management

☐ Acne-related scar prevention/management

☐ Striae (stretch mark) prevention/management

☐ Traumatic wound-related scar prevention/management (e.g., abrasions, diabetic ulcers, pressure ulcers)

☐ Herpes zoster, varicella, or HSV-related scar prevention/management

☐ Scar prevention/management following skin necrosis from filler complications

☐ Scar prevention/management after energy-based device (EBD) procedures, especially ablative lasers

**Section I. PN Use and Combination Approaches in Post-Surgical Scars**

(Follow-up questions for those who selected "post-surgical scar prevention/management" in H1)

I1. In which post-surgical scenarios would you consider applying PN?

(Select all that apply)

☐ Immediately before/after surgery for scar prevention (e.g., to prevent prolonged erythema or raised scarring)

☐ For atrophic surgical scars

☐ For hypertrophic surgical scars

**Section I-a. Scar Prevention After Surgery**

I-a-1. What agents would you recommend combining with PN for surgical scar prevention?

(Select all that apply)

☐ None

☐ Fillers (crosslinked HA, non-crosslinked HA, collagen, etc.)

☐ Injectable biostimulators (PLA, PMA, PDO, PCL, CaHA, etc.)

☐ Botulinum toxin

☐ Topical skin boosters (e.g., exosomes, stem cell culture media, growth factors such as EGF, FGF, IGF, or cosmetic biostimulators)

☐ Tissue regenerative agents (e.g., PRP, PDRN)

☐ Anti-fibrotic agents (e.g., steroid, 5-FU, bleomycin)

☐ Other (please specify): ___________

I-a-2. What devices would you recommend combining with PN for surgical scar prevention?

(Select all that apply)

☐ None

☐ Vascular lasers (e.g., PDL, long-pulsed Nd:YAG [1064 nm], KTP)

☐ Pigment lasers (e.g., Q-switched Nd:YAG [532, 1064 nm], Q-switched ruby, Alexandrite, picosecond lasers)

☐ Ablative fractional lasers (e.g., fractional CO₂, fractional Er:YAG)

☐ Non-ablative fractional lasers (e.g., Er:Glass, fractional picosecond [532/1064 nm], thulium)

☐ Non-invasive radiofrequency devices (e.g., Thermage)

☐ Invasive microneedle RF devices

☐ Cryotherapy devices

☐ Pneumatic (needle-free jet) injectors

☐ Low-level laser therapy (e.g., LED, HeNe laser)

☐ Other (e.g., microcoring, ESWT, plasma, IPL): ___________

**Section I-b. PN Use and Combination Approaches for Atrophic Post-Surgical Scars**

(Follow-up for these who selected "Atrophic scar management" in I1)

I-b-1. What agents would you recommend combining with PN for atrophic surgical scars?

(Select all that apply)

☐ None

☐ Fillers (crosslinked HA, non-crosslinked HA, collagen, etc.)

☐ Injectable biostimulators (PLA, PMA, PDO, PCL, CaHA, etc.)

☐ Botulinum toxin

☐ Topical skin boosters (e.g., exosomes, stem cell culture media, growth factors such as EGF, FGF, IGF, or cosmetic biostimulators)

☐ Tissue regenerative agents (e.g., PRP, PDRN)

☐ Anti-fibrotic agents (e.g., steroid, 5-FU, bleomycin)

☐ Other (please specify): ___________

I-b-2. What devices would you recommend combining with PN for atrophic surgical scars?

(Select all that apply)

☐ None

☐ Vascular lasers (e.g., PDL, long-pulsed Nd:YAG [1064 nm], KTP)

☐ Pigment lasers (e.g., Q-switched Nd:YAG [532, 1064 nm], Q-switched ruby, Alexandrite, picosecond lasers)

☐ Ablative fractional lasers (e.g., fractional CO₂, fractional Er:YAG)

☐ Non-ablative fractional lasers (e.g., Er:Glass, fractional picosecond [532/1064 nm], thulium)

☐ Non-invasive radiofrequency devices (e.g., Thermage)

☐ Invasive microneedle RF devices

☐ Cryotherapy devices

☐ Pneumatic (needle-free jet) injectors

☐ Low-level laser therapy (e.g., LED, HeNe laser)

☐ Other (e.g., microcoring, ESWT, plasma, IPL): ___________

**Section I-c. PN Use and Combination Approaches for Hypertrophic Post-Surgical Scars**

(Follow-up for those who selected "Hypertrophic scar treatment" in I1)

I-c-1. What agents would you recommend combining with PN for hypertrophic surgical scars?

(Select all that apply)

☐ None

☐ Fillers (crosslinked HA, non-crosslinked HA, collagen, etc.)

☐ Injectable biostimulators (PLA, PMA, PDO, PCL, CaHA, etc.)

☐ Botulinum toxin

☐ Topical skin boosters (e.g., exosomes, stem cell culture media, growth factors such as EGF, FGF, IGF, or cosmetic biostimulators)

☐ Tissue regenerative agents (e.g., PRP, PDRN)

☐ Anti-fibrotic agents (e.g., steroid, 5-FU, bleomycin)

☐ Other (please specify): ___________

I-c-2. What devices would you recommend combining with PN for hypertrophic surgical scars?

(Select all that apply)

☐ None

☐ Vascular lasers (e.g., PDL, long-pulsed Nd:YAG [1064 nm], KTP)

☐ Pigment lasers (e.g., Q-switched Nd:YAG [532, 1064 nm], Q-switched ruby, Alexandrite, picosecond lasers)

☐ Ablative fractional lasers (e.g., fractional CO₂, fractional Er:YAG)

☐ Non-ablative fractional lasers (e.g., Er:Glass, fractional picosecond [532/1064 nm], thulium)

☐ Non-invasive radiofrequency devices (e.g., Thermage)

☐ Invasive microneedle RF devices

☐ Cryotherapy devices

☐ Pneumatic (needle-free jet) injectors

☐ Low-level laser therapy (e.g., LED, HeNe laser)

☐ Other (e.g., microcoring, ESWT, plasma, IPL): ___________

**Section J/K/L/M/N/O/P Repetition of Section I for other scar categories.**
